# The Impact of Step Reduction on Muscle Health in Aging: Protein and Exercise as Countermeasures

**DOI:** 10.3389/fnut.2019.00075

**Published:** 2019-05-24

**Authors:** Sara Y. Oikawa, Tanya M. Holloway, Stuart M. Phillips

**Affiliations:** Exercise Metabolism Research Group, Department of Kinesiology, McMaster University, Hamilton, ON, Canada

**Keywords:** step reduction, physical inactivity, protein, muscle, older adults

## Abstract

Declines in strength and muscle function with age—sarcopenia—contribute to a variety of negative outcomes including an increased risk of: falls, fractures, hospitalization, and reduced mobility in older persons. Population-based estimates of the loss of muscle after age 60 show a loss of ~1% per year while strength loss is more rapid at ~3% per year. These rates are not, however, linear as periodic bouts of reduced physical activity and muscle disuse transiently accelerate loss of muscle and declines in muscle strength and power. Episodic complete muscle disuse can be due to sickness-related bed rest or local muscle disuse as a result of limb immobilization/surgery. Alternatively, relative muscle disuse occurs during inactivity due to illness and the associated convalescence resulting in marked reductions in daily steps, often referred to as step reduction (SR). While it is a “milder” form of disuse, it can have a similar adverse impact on skeletal muscle health. The physiological consequences of even short-term inactivity, modeled by SR, show losses in muscle mass and strength, as well as impaired insulin sensitivity and an increase in systemic inflammation. Though seemingly benign in comparison to bed rest, periodic inactivity likely occurs, we posit, more frequently with advancing age due to illness, declining mental health and declining mobility. Given that recovery from inactivity in older adults is slow or possibly incomplete we hypothesize that accumulated periods of inactivity contribute to sarcopenia. Periodic activity, even in small quantities, and protein supplementation may serve as effective strategies to offset the loss of muscle mass with aging, specifically during periods of inactivity. The aim of this review is to examine the recent literature encompassing SR, as a model of inactivity, and to explore the capacity of nutrition and exercise interventions to mitigate adverse physiological changes as a result of SR.

## Physical Activity and Aging

In Canada, ~85% of individuals are not meeting physical activity guidelines (1). This highlights the potential for improvement that could be achieved given the potential for increased physical activity to reduce risk for a number of diseases and for all-cause mortality (2, 3). Older adults tend to engage in less physical activity in comparison to younger adults (4) with a notable decline in levels of leisure time physical activity in older adults (5–7). Interestingly, social isolation in older persons may be result from numerous factors: inability to leave the house due to poor mobility, lack of transportation, or adverse weather conditions, illness of the individual or in their social circles, all of which highlight the complexity for the capacity of intervention in aging adults.

Exacerbating low levels of habitual physical activity in older adults are abrupt and acute reductions in activity resulting in lower levels of mechanical loading of muscle. Acute bouts of inactivity that result in unloading of muscles manifest due to a variety of circumstances (illness, injury, poor weather conditions) and are distinctly different from habitual sedentary behavior. Though these acute disruptions in activity may be seemingly benign, we hypothesize that accumulated bouts of marked inactivity superimposed on a physically inactive population is a major risk for negative physiological health outcomes and may accelerate sarcopenia and the development of chronic cardiometabolic conditions associated with aging.

### Sarcopenia and Physical Inactivity

**Figure 1:**
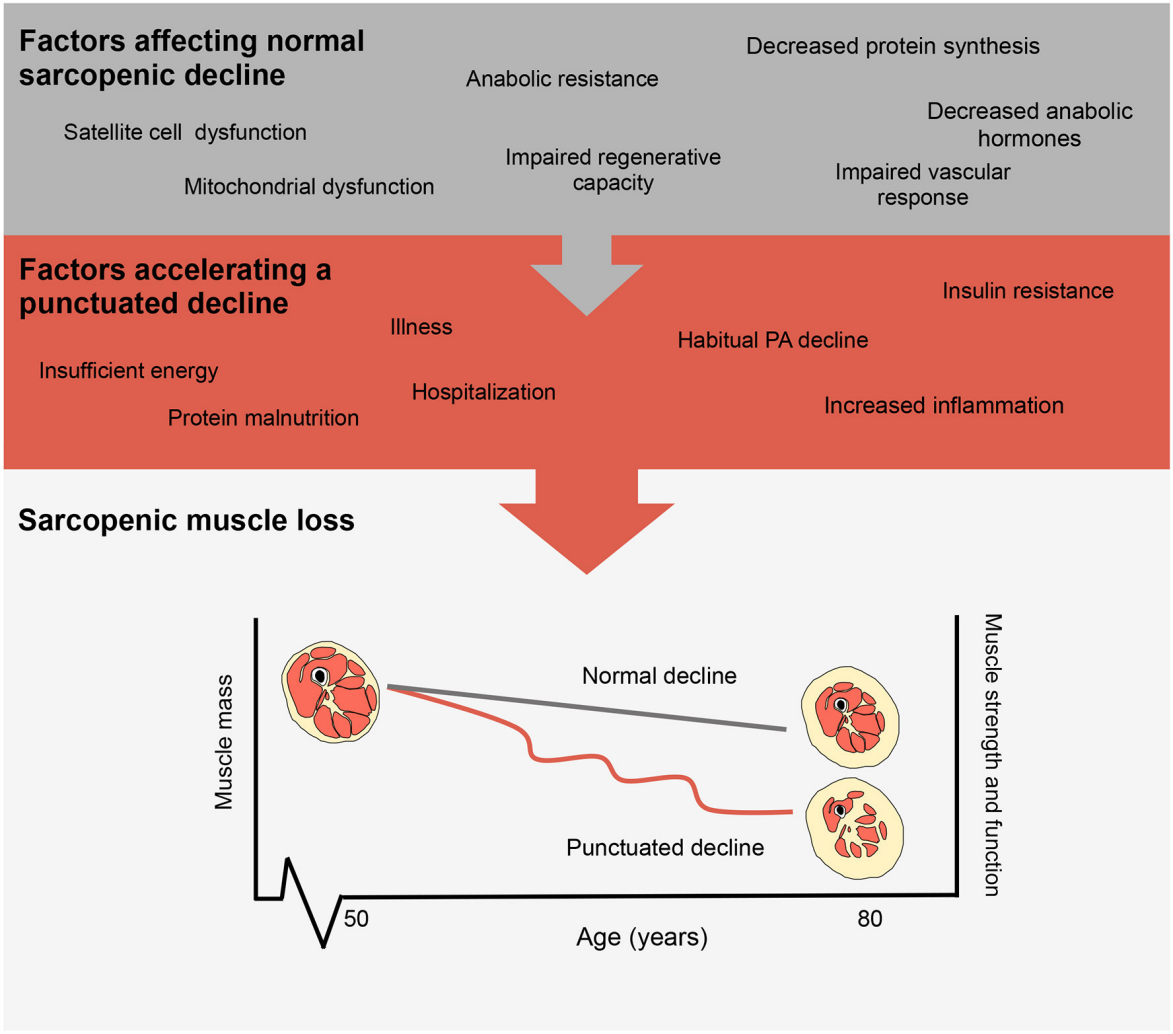
Factors influencing the progression of sarcopenia. Representations of normal sarcopenic muscle loss and accelerated muscle loss as shown by a punctuated decline.

Cyclical bouts of pronounced inactivity, even in relatively healthy persons, can have significant detrimental physiological effects on health particularly with advancing age (8). Specifically, acute periods of physical inactivity (9–14) lead to reductions in skeletal muscle size and strength that transiently expedite the usual declines resulting from sarcopenia (15). Population-based estimates of sarcopenia show muscle loss occurring at a rate of ~1% per year with losses in muscle strength and power, more rapid at rates of ~3% and ~8% per year, respectively, (16, 17). Though the progression of sarcopenia is seen as a normal consequence of aging it can be accelerated due to inactivity, which transiently accelerates muscle loss (15). Indeed, numerous factors can affect the progression of sarcopenic muscle loss with inactivity events further accelerating muscle loss as shown in Figure 1. Lifestyle factors such as exercise and nutrition may moderate the progression of normal muscle loss with increasing age. In particular, declines in physical activity, insufficient or excess energy intake, and protein malnutrition may act to synergistically accelerate sarcopenic declines and thus increase the risk for subsequent hospitalization or disuse resulting in accelerated muscle loss (18). Importantly with each disuse event, muscle mass loss decreases and muscle cross sectional area is drastically reduced with an increase in intramuscular fat content (19). Physical activity is a potent regulator of factors associated with aging and skeletal muscle health [inactivity and inflammation (14), reactive oxygen species, glycemic control (20), loss of motor neurons (21)] and when combined with proper nutrition (adequate protein intake) may serve to attenuate the rate of muscle decline.

## Step Reduction as a Model of Periodic Inactivity

**Figure 2:**
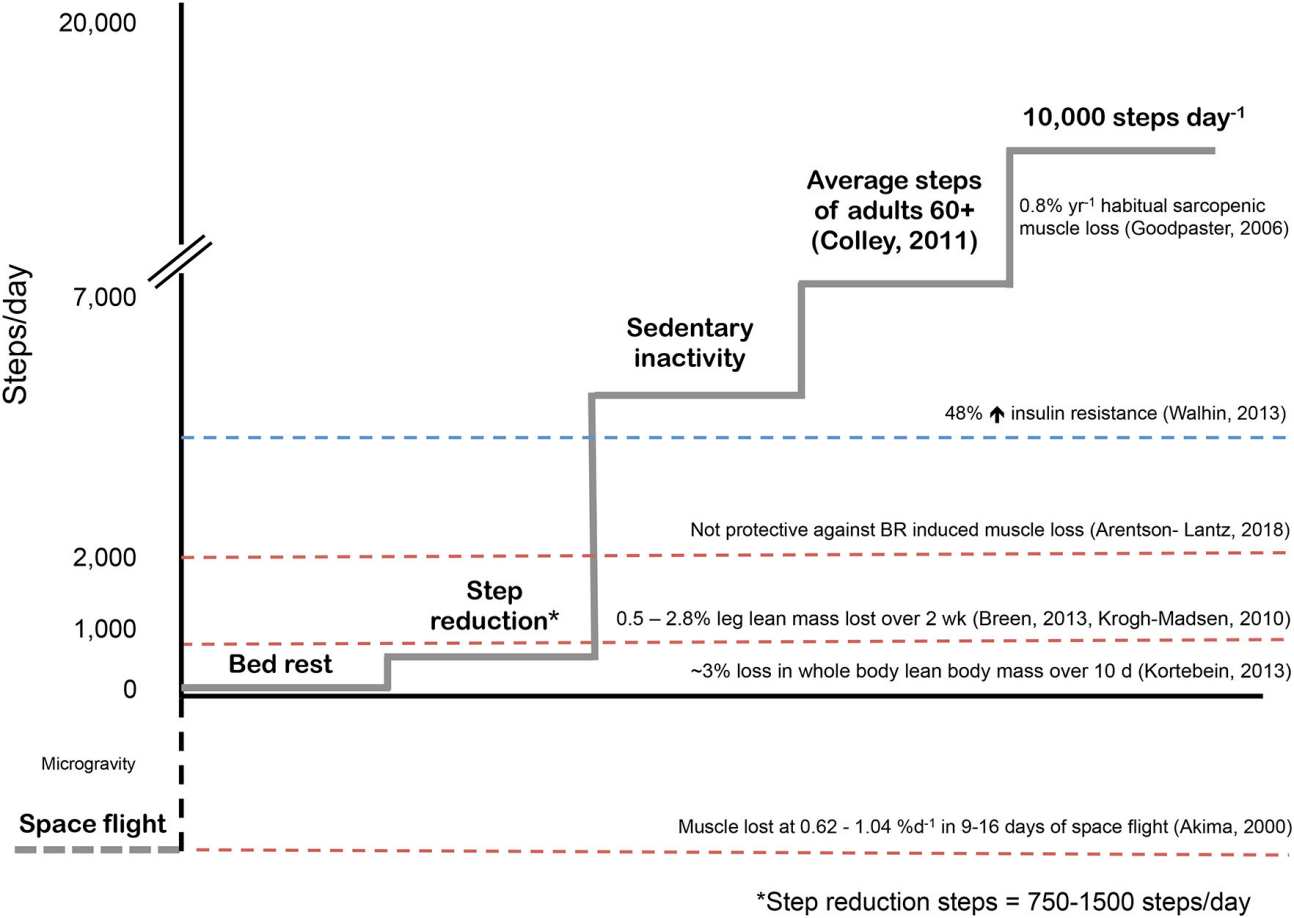
Physical inactivity models used in human skeletal muscle metabolism. Typical sarcopenic muscle loss based on population estimates (24). Sedentary behavior as categorized by Walhin et al. as < 4000 steps per day induced significant insulin resistance in healthy young adults (25). Interestingly, during one week of bed rest, participants walked for ~22 minutes per day (~2000 steps) in an effort to offset bed rest induced muscle atrophy however exercise was not able to mediate this effect and muscle loss was similar to controls (26). Step reduction, or abruptly reducing habitual daily steps to 750-1500 steps per day results in leg lean mass loss over two weeks in healthy young and older adults (13, 14, 27). Bed rest induces rapid muscle atrophy in healthy older adults (9) however the rate of muscle loss per day as a result of space flight or exposure to microgravity are staggering (28).

**Figure 3:**
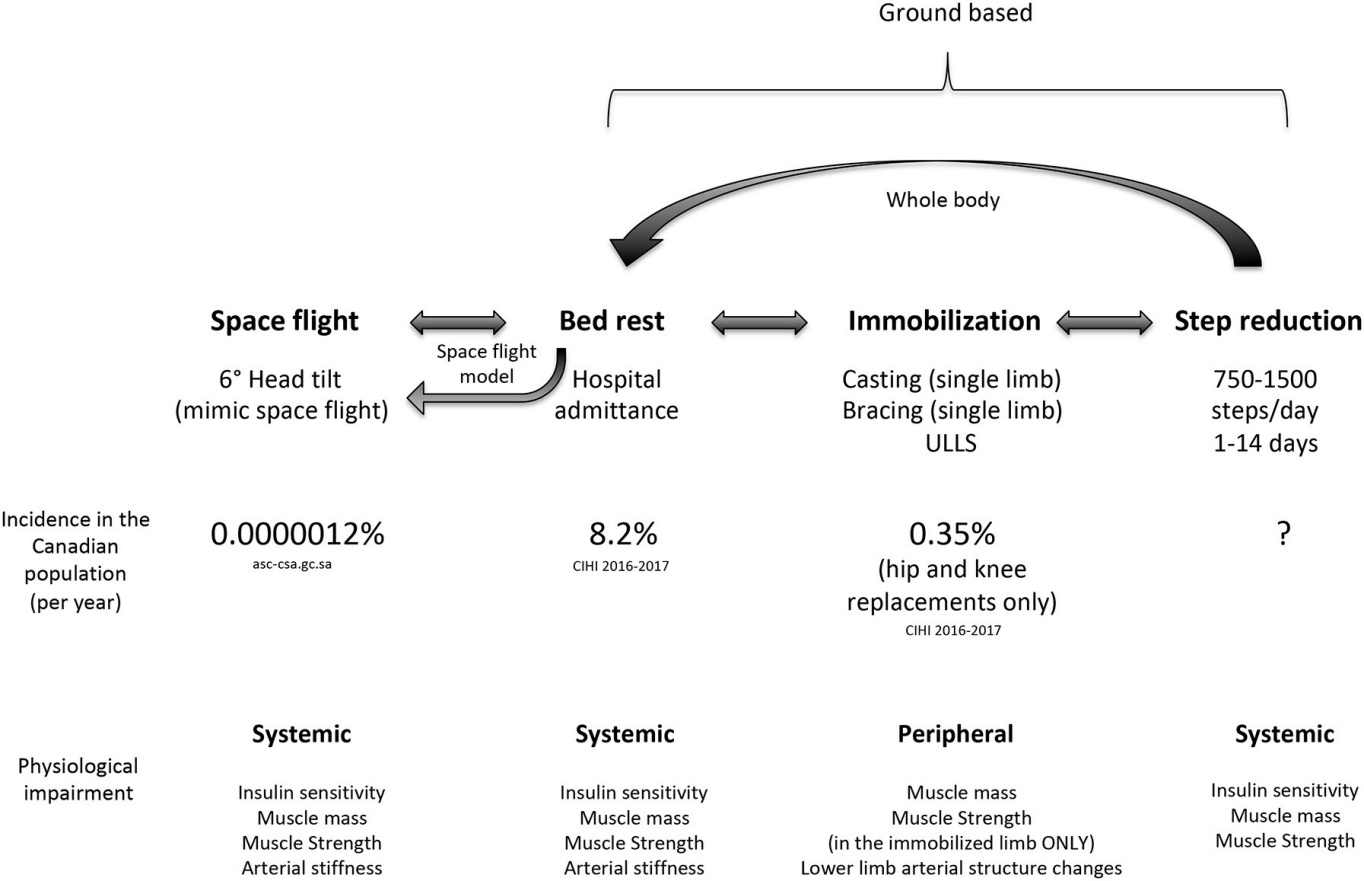
Comparison of disuse models used to study inactivity highlighting the whole body systemic nature of bed rest and step reduction compared to limb immobilization. Data regarding the prevalence of events with induced step reduction is currently unknown.

Previous studies have employed various models to study physical inactivity in humans ranging from a brief reduction in habitual physical activity (90% reduction in daily steps for 1 day) (22) to spaceflight and microgravity (23). As shown in Figure 2, each reduced activity model results in differences in daily activity level, which are notably reduced from habitual physical activity levels in older adults. Inactivity during bed rest has provided researchers with a characteristic change in muscle phenotype in order to better understand the physiological consequences of disuse (29). Given that bed rest requires inactivity of the whole body, it provides an excellent model to understand the systemic effect of disuse on multiple physiological systems and is clinically relevant (30). Conversely, single limb immobilization studies in older (11, 12) and younger (11, 31, 32) adults have emphasized the significant physiological consequences occurring with local muscle-level disuse. Immobilization-induced muscle loss is largely applicable to clinical scenarios of single-limb immobilization or elective orthopedic surgery, during which the recovery of the affected limb may be without loading for several weeks (11, 12, 20). More recently, the investigation of SR as a form of abrupt physical inactivity has been employed, to investigate the effects of abrupt reductions in activity but not complete disuse. During SR, participants are asked to reduce their daily steps (usually externally monitored by a pedometer or similar device) to a low maximal daily step count (750–5,000 steps/d) (14, 27, 33). The lower end of daily step count (~750 steps/d) used in studies is in line with steps performed by patients in acute hospital stays (34). Alarmingly, the daily steps of patients in hospital (out of 708 days examined) exceeded 300 steps per day only 50% of the time; however, on average daily steps per patient were ~740 (34). Reductions of physical activity with SR to these low levels would not obviously constitute complete muscle disuse, but do have profound physiological consequences. Importantly, SR has similar whole body systemic effects, but obviously to a lesser degree, as bed rest, in comparison to unilateral limb immobilization which largely targets peripheral tissues (Figure 3). Additionally, episodes of bed rest (typically due to hospital admittance) arguably occur less frequently than episodes of inactivity that occur periodically throughout due to weather or illness such as influenza and likely affect a greater proportion of the population than is affected by complete bed rest. Thus, the purpose of this review is to highlight the physiological consequences resulting from purposeful reduced daily steps in younger and older adults with insight on recent studies using SR and the potential for exercise and nutrition to combat disuse atrophy in this model.

### Physiological Consequences of SR in Young Adults

The first study establishing the physiological consequences of SR was by Olsen et al. who elegantly demonstrated that reducing daily steps for as little as 3-wks had marked negative impacts on skeletal muscle (35). Participants (young healthy adults) reduced their step count by simply taking the elevator instead of stairs and utilizing cars instead of walking or bicycling resulting in daily average steps totalling ~1,300 steps/d (reduced from a habitual step count of ~10000 steps/d). Following 21 days of SR, participants had decreased insulin sensitivity, attenuation of postprandial lipid metabolism, and increased intra-abdominal fat mass (35). The negative alterations in glycemic control and impaired lipid metabolism (35) highlight the rapidity of how healthy and mobile individuals move to metabolic dysfunction simply through a period of SR as well as how easily, through alterations in use of personal and publication transportation that adults can reduce daily step count.

Knudsen et al. also demonstrated that reducing daily steps from 10,000 to 1500, in combination with over-feeding, increased visceral adiposity by 49% and decreased insulin sensitivity by 44% in healthy, active, young adults (36). Likewise, Krogh-Madsen et al. showed that reducing daily physical activity to levels similar to the previously described intervention but maintaining energy balance (36), resulted in a reduced insulin sensitivity, a reduction in VO_2_ max of 7 mL/kg/min and a 0.5 kg decrease in leg lean mass (LLM) in healthy young men (27). Taken together, data from Knudsen et al. (36), and Krogh-Madsen et al. (27), demonstrate the potency of a SR model and the susceptibility of healthy young adult populations to significant negative metabolic health outcomes following even brief periods of inactivity.

Interestingly, Stephens et al. (22) showed that even 1 day of limited physical activity (~260 steps) was a sufficient stimulus to induce a impairments in insulin action, as measured by whole body rate of glucose disappearance, in physically active, young men and women by ~39%. Though the steps per day in that investigation (22) were low for a healthy adult, when viewed in the context of severe illness or hospital stay, the daily step counts are on par with those of hospitalized patients (34). Further, given that the impairments to reduced steps in young healthy adults are notably adverse, then it is not unreasonable to hypothesize that the effects would be of greater magnitude in a compromised aging population (18). Nevertheless, measures to mitigate rapid declines in physical activity even in young adults should be further explored in order to offset the negative physiological adaptations to SR.

### Physiological Consequences of SR in Older Adults

Older adults, compared to their younger counterparts, may be at a greater risk for periods of inactivity in addition to a declines in habitual physical activity (4). In addition to the frequency of periodic SR, the adverse consequences of such periods of inactivity in older persons are also likely much greater than they are in younger persons. For example, in a seminal study by Suetta et al. (11), the authors found that following 2-weeks of unilateral leg casting and subsequent intensive resistance training that older adults were not able to fully recover losses of skeletal muscle in comparison to a cohort of young adults who demonstrated full recovery of quadriceps cross sectional area (CSA) (11). Similarly, healthy older adults have shown marked susceptibility to acute reductions in daily stepping impacting glycemic control, markers of inflammation, and skeletal muscle, the recovery from which is incomplete following SR, underlining an obvious impaired regenerative capacity in aging skeletal muscle (20).

### Glucose Handling and Inflammation With SR in Older Adults

Similar to healthy young adults, healthy older adults also demonstrate negative effects on glucoregulation with SR. McGlory et al. (20), examined how pre-diabetic older adults responded metabolically to 2 weeks of SR and importantly whether participants were able to recover to pre-SR levels. In this study (20), participants reduced their daily steps to <1000 steps/d followed by a two-week recovery period in which they returned to habitual levels of activity while maintaining energy balance. Fasted plasma glucose and insulin levels were significantly elevated following SR by 8 and 31%, respectively and these values did not return to pre-SR levels following a two-week recovery (20). Comparably, Breen et al. had participants reduce their daily step count by ~75% or to ~1500 steps/day for 2 weeks and monitored changes in glycemic control. Though the authors found no significant elevation in fasted blood glucose following SR, they noted a significant elevation in fasted plasma insulin as well as an increase in glucose and insulin area under the curve by 9 and 12%, respectively confirming an impaired glycemic handling in response to SR (13). It is notable that in both studies (13, 20), that SR resulted in small but significant increases in systemic inflammatory cytokines. Breen et al. observed elevated levels of TNF- α and CRP in response to inactivity in normoglycemic participants while McGlory et al. observed an increase in levels of TNF-α, IL-6 and CRP following SR in overweight and obese pre-diabetic participants. The concentrations of inflammatory markers were partially recovered following return to habitual activity in pre diabetic participants (20). Interestingly, older adults showed a rise in inflammatory cytokines (13, 20), which is something that was not seen in younger adults in response to SR (27, 36). While we acknowledge that this is an observation, we posit that this may be of some significance in explaining why younger persons do and older persons do not recover from SR (37); however, this would require specific examination.

### Changes in Muscle Protein Turnover and Muscle With SR

Previous work from our group (13, 20, 38) and others (39, 40), have attributed the loss of muscle mass with muscle disuse, for the most part, to a reduction in both fasted- and fed-state muscle protein synthesis (MPS) (41). Indeed, alterations in skeletal muscle protein synthesis are highly sensitive to modifications in physical activity and mechanical loading to similar extent in both younger and older adults (9, 31). To date, no studies have examined the impact of SR on modifications in MPS in younger adults in response to SR. Nonetheless, Krogh-Madsen et al. (27) did observe a loss of leg lean mass of 2.8% following 2 weeks of reduced daily stepping (<1500 steps per day), an observation that emphasizes the impact of SR in healthy young adults. Several studies have investigated the effects of SR on losses in lean body mass (LBM) and modifications in rates of MPS in older adults (13, 14, 20, 38). Consistent with the concept of muscle disuse-induced “anabolic resistance” (12, 31) work from our laboratory has shown consistent reductions in MPS in response to 2 weeks of SR of varying degrees (750–1500 steps per day) (13, 14, 20) with rates reduced 13–26% from baseline. Importantly, in healthy older adults McGlory et al. demonstrated that in the absence of rehabilitative measures (i.e., additional physical activity/loading above baseline levels, nutritional intervention) the reduced rates of MPS seen during inactivity were not recovered following 2 weeks of return to habitual activity (17).

Given that MPS is a strong regulator of skeletal muscle mass in healthy populations (42), strategies to improve MPS in response to SR may prove to be promising in the maintenance of skeletal muscle during and in recovery from acute inactivity due to illness. Importantly, older adults typically have less muscle mass in comparison to younger adults (43) when they become inactive. While the absolute loss of skeletal muscle in older adults in response to reduced activity models may be less (11) the relative loss is significantly greater than losses in younger adults in bed rest protocols (44). Nonetheless, the lack of recovery seen in older as opposed to younger adults is a troubling observation (11, 20).

### Changes in Muscle Function and Physical Capacity With SR

The association of skeletal muscle mass and skeletal muscle strength has been well-established, where reductions in muscle mass/area are roughly correlated with reductions in muscle strength and power (24, 45). Low skeletal muscle strength (but not mass) is an independent risk factor for mortality (46). Thus, determining the impact of seemingly benign periods of reduced daily activity, via models such as SR, on skeletal muscle functional outcomes is imperative, specifically for older adults for whom losses in strength have a substantial effect on quality of life (47).

As mentioned previously, young adults exhibit notable decrements in maximal aerobic capacity [3–7% (27, 36)] following SR (<1500 steps per day) lasting only 14 days. Evidence substantiating alterations in maximum voluntary strength of the lower limbs in response to SR in older adults have been varied (14, 17, 38). Reidy et al. found that knee extensor maximum voluntary contraction (MVC) was significantly reduced by ~8% in older adults during moderate SR (<3,000 steps per day, 2 weeks) (48) while we observed a reduction in MVC of 9% and 6% in men and women, respectively, following SR (<750 steps per day, 2 weeks) (49). Further in the investigation by Reidy et al. strength losses were not recovered (48) after 14 days of return to normal activity and were only recovered in men in work by Oikawa et al. (49). Conversely, reductions in knee extensor MVC were not observed in previous investigations of SR despite marked metabolic and physiological perturbations in glucose regulation, inflammation and reductions in MPS (13, 20, 38); however, we speculate that differences in familiarization procedures might underpin this heterogeneity of response. Substantial familiarization is required in order to obtain a true baseline strength measurement especially in older persons (50), along with the small changes in strength expected with SR (compared to complete disuse) may be responsible for the observed heterogeneity in MVC as measured by dynamometry following SR. To date, no study has examined the impact of SR on strength or clinical functional parameters in healthy young adults though these outcomes appear to be preserved in older adults following SR, unlike the decrements observed in models of complete unloading (bed rest) (9, 51). Though the decrements in muscle strength reported by Reidy and Oikawa are small, it should be acknowledged that a lack of strength recovery poses a significant threat in the progression of healthy aging. Without recovery of lost strength, each future perturbation in physical activity will reduce an individual's maximal strength output increasing risk for disability and mobility impairments (47). Thus, strategies to restore muscle strength and function following SR are imperative in order for maintenance of independence and quality of life throughout aging.

## Mitigating the Physiological Consequences of Disuse and SR With Exercise

Muscular contraction is a potent stimulus to attenuate the negative effects of muscle disuse on skeletal muscle loss (38, 52). Resistance training (RT) has been shown to increase skeletal muscle mass (53, 54), capillary density (55), and satellite cell activation in older adults (56) making it an obvious countermeasure to combat skeletal muscle atrophy.

Previous literature employing resistance exercise to offset declines in LBM during bed rest have been successful (52, 57–59). Bamman et al. found significant decreases in Type I and II fiber CSA while myofibre CSA was maintained in the exercise and bed rest group of young men (57) while Kawakami et al. found that exercise attenuated the decline in muscle CSA as measured by magnetic resonance imaging (MRI) in young men (58). Similarly, Alkner et al. showed that following 90 days of bed rest that a RT group showed no decrease in total quadriceps muscle volume while the non-exercise control group showed a decrease decreased of ~18% (52). Trappe et al. showed that whole quadriceps muscle volume as measured by MRI decreased by ~17% while there was no decline in muscle volume in the RT group after 84 days of bed rest (59). Oates et al. also showed that even a very low volume of RT performed every other day was sufficient to mitigate declines in muscle CSA of the triceps surae and knee extensors (60).

To date, only one study has been conducted in which periodic low-level resistance exercise has been used to offset SR-induced muscle atrophy (38). Devries et al. utilized a unilateral model of resistance training during SR in which older participants were asked to reduce their daily step count to <1500 steps per day for 2 weeks (38) and performed unilateral low-load resistance exercise at 30% of their maximal strength (~20-25 repetitions) three times per week. Low load RT has significant promise to induce skeletal muscle hypertrophy and even strength (61, 62) and could possibly be useful in situations where high load exercises are not possible such as hospitalization or when home bound due to illness. Following 2-weeks of SR, the leg that performed RT was protected against the SR-induced reduction in postabsorptive and postprandial MPS seen in the non-exercised SR leg (38). Data from the same group of participants was analyzed for alterations in satellite cell activation in both the SR and SR plus RT limbs and found that RT was effective at preserving Type I and II fiber cross sectional area, similar to findings during bed rest (57), and in the preservation of Pax7^+^ positive cells (satellite cells) in type I and II fibers (63), which were lower in the SR leg. Given the robust impact of resistance exercise on skeletal muscle anabolism in younger and older adults (56, 61) it is not wholly surprising that RT was able to preserve aged skeletal muscle during SR to levels similar to healthy controls. Though the applicability of these data (38) is debatable since we recognize that older adults who are taking <1500 steps per day may not be able to perform RT if the nature of the inactivity is caused by illness or injury. Nonetheless, we propose that the potentially favorable effects that even infrequent low load muscular contractions may have on the preservation of skeletal muscle health should be considered in an effort to reduce muscle mass and strength loss with disuse. Interestingly, a study by Arentson-Lantz et al. (26), aimed to determine whether taking 2000 steps/d (~22 min of walking per day) during 1 week of bed rest would be a sufficient stimulus to offset losses in skeletal muscle mass and physical function. Following 1 week, participants lost ~1 kg of LLM with no effect of the added daily stepping, findings that were confirmed by immunohistochemistry determining fiber CSA. Further, increased daily stepping did not attenuate the reduction in leg MVC, with participants exhibiting strength losses of ~12% (26). This study provides compelling data, highlighting the powerful effects that complete bed rest can have. Indeed, strategies to reduce the impact of bed rest and disuse are imperative to the conservation of skeletal muscle and functional outcomes in older adults.

### The Role for Nutrition in Attenuating LBM Losses With Disuse

**Figure 4:**
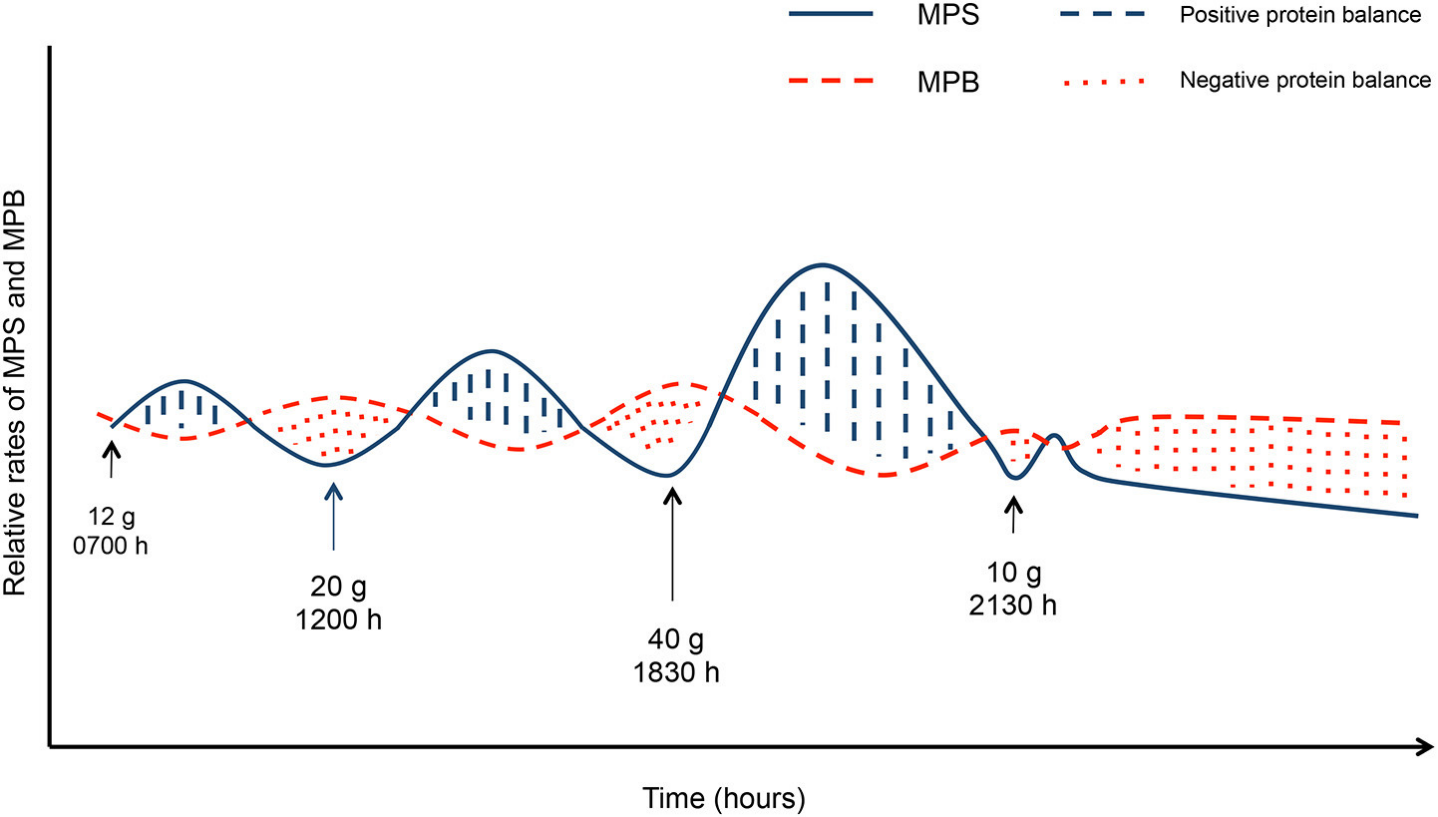
Muscle protein synthesis (MPS) and muscle protein breakdown (MPB) in responses to grams of protein per meal. Solid lines indicate MPS, dashed lines indicate MPB. Blue hashed areas indicate positive protein balance while the red dotted areas indicate negative protein balance. Blue hashed areas and red dotted areas equate to the same area under the curve indicating net protein balance.

The term anabolic resistance describes the reduction in MPS in response to a given protein dose (64) (and subsequent hyperaminoacidemia) and to a bout of resistance exercise with age (65, 66) and is a negative consequence induced by disuse (31). The role of nutrition to mitigate the negative physiological consequences of inactivity has been examined largely in the context of bed rest (40, 51, 67–69) and single leg immobilization (12, 70) with a majority of studies examining protein or amino acid based supplements (40, 69, 71, 72). Supplementation with amino acids may represent an effective strategy to combat both anabolic resistance with age and anabolic resistance as a result of disuse, particularly if the supplemental protein or amino acids are used to create a more even meal-to-meal pattern with protein intake at each meal (73, 74). This is largely because adults tend to consume the largest amounts of protein; amounts sufficient to induce maximal rates of MPS only at the later meals of the day (75) as shown in Figure 4 and thus the addition of a protein supplement at meals throughout the day may serve to facilitate a better stimulation of MPS and positive net protein accretion (74).

The essential amino acid leucine is a potent stimulator of mTOR and in turn, MPS (76) and therefore, supplements high in leucine content are typically used for their anabolic potential and potential muscle sparing effect during disuse atrophy. To date however, results have been incongruent in bed rest models. Ferrando et al. found that supplementation with 15 g of essential amino acids (5.3 g of leucine) thrice daily, during 10 days of bed rest did not alleviate LBM loss in healthy older adults in comparison to a control group (51). Similarly, English et al. found that meal time supplementation with doses of 4.5 g of leucine only partially protected LBM loss after 7 days of bed rest but did not significantly protect LBM at 14 days of bed rest with supplementation in younger adults (40). Paddon-Jones et al. showed a protective effect of 16.5 g of EAA (3.1 g of leucine) provided three times daily, during 28 days of bed rest on total LBM in young adults (72). Given that the lack of agreement on the efficacy of amino acid supplementation on the sparing of LBM is in both young and older adults during bed rest, much more research is needed in order to definitively determine whether there is indeed a benefit of EAA and leucine supplementation during bed rest.

Interestingly, energy balance appears to play a significant role on LBM loss during bed rest. As might be expected, consumption of a hypocaloric diet results in an accelerated rate of LBM loss during bed rest, largely through suppression of MPS (42). Indeed, Biolo et al. showed that 14 days of bed rest in combination with a 20% caloric deficit led to the greater wasting of LBM compared to the same participants consuming a eucaloric diet in a cross-over study design (68). However, in a subsequent study, Biolo et al. also examined the effects of positive energy balance during bed rest in comparison to negative energy balance. These authors found that during 35 days of bed rest, participants in positive energy balance, lost 1.5 kg more LBM than participants in negative energy balance, a finding that the authors attributed to an activation of inflammatory pathways associated with the increase in fat mass accompanying the positive energy balanced state. Thus, in addition to nutrient supplementation, a consideration should be made to encourage maintenance of energy balance during periods of disuse for optimal nutrition to attenuate skeletal muscle loss.

Fewer studies have examined the effects of nutritional interventions during single limb immobilization in humans. Two studies have examined the effects of creatine supplementation with mixed results. Hespel et al. examined the effects of daily supplementation with 20 g of creatine monohydrate or placebo control for 2 weeks of unilateral lower limb (knee) immobilization in young adults. These authors found a significant loss in quadriceps muscle CSA with no difference between supplemental groups (77). Conversely, Johnston et al. provided young adult participants with both a placebo control and subsequently 20 g of creatine during 7 days of upper limb (elbow joint) immobilization in a cross-over design study and creatine better maintained lean tissue mass compared to the placebo while also maintaining a variety of functional parameters (78). Though there are many differences between the two aforementioned studies (immobilization time, measurement of muscle mass), the difference in immobilized limb (weight bearing vs. non-weight bearing) makes it difficult for comparison between the two studies and thus the effect of creatine supplementation on LBM retention with immobilization requires further investigation.

Protein and amino acid supplementation has not been widely examined in the literature to offset muscle loss during immobilization. Dirks et al. showed that following 5 days of cast immobilization, quadriceps CSA was reduced by 1.5% in controls and by 2% in healthy older men consuming a placebo or twice daily 20.7 g protein supplement, respectively, (12). Interestingly participants in this study were provided with a protein supplement that may have been below optimal thresholds [0.4 g/kg/dose (79)] as based on average mass a protein dose closer to 30 g may have been more effective to attenuate the loss of LBM with cast immobilization of the knee (79).

Recently, McGlory et al. examined the effect of fish oil supplementation on the retention of LBM during unilateral knee bracing for two weeks in young healthy women. Participants were provided with a daily dose of 5 g of n-3 fatty acids or a control oil (sunflower oil) for 28 days prior to 14 days of unilateral knee immobilization. Following immobilization, supplementation with n-3 fatty acids attenuated the decline in quadriceps muscle volume by 6% (14 vs. 8% in the control vs. supplement group, respectively) and also promoted an elevation of MPS above rates of the control group at all measured time points (70). Thus, the addition of n-3 fatty acid supplementation in addition to an adequate protein dose and energy balance may serve to attenuate the decline in LBM during unilateral limb immobilization and bed rest.

To date no study has examined the effect of dietary manipulations on SR through supplementation or changes in energy intake. Given that protein, specifically, high quality protein (high in EAA) in large quantities has a potent anabolic effect on older skeletal muscle, we recently aimed to examine the effects of a high protein diet to mitigate skeletal muscle loss and reductions in MPS in healthy older adults during SR. To recapitulate circumstances associated with a period of SR in older adults (i.e., hospitalization), participants were provided with a high protein (1.6 g/kg/day) by supplementation with either a 60 g daily dose of whey protein or collagen peptides, and an energy restricted diet (−500 kcal, in addition to the reduction in energy intake accounted for by the SR) during a 2-week SR period. Supplementation with whey protein or an isonitrogenous quantity of hydrolyzed collagen peptide supplement allowed for the comparison of supplementation with high and low quality, high protein on LBM changes with SR (14). Following 1 week of energy restriction alone, there were no significant losses in LBM, however there was a marked reduction in MPS of ~16% in both groups [findings similar to previous investigations in SR (20)]. Interestingly, with energy restriction and SR, there were no further reductions in MPS, indicating a potential protective mechanism by which healthy older adults are able to mitigate additive catabolic stimuli. However, following a 1 week recovery which featured a return to normal daily steps, during which participants maintained supplementation, whey protein proved to be superior in stimulating rates of MPS above the SR period with no effect of collagen peptide supplementation (14). The lack of recovery of rates of MPS in the collagen supplemented group following return to normal activity is similar to previous findings from our laboratory in which participants resumed consumption of their habitual diets after SR (20). These data highlight the promising observation that high quality protein supplementation may serve to improve skeletal muscle health in conjunction with an increase in physical activity in older adults following convalescence, results that could be possibly further enhanced when combined with structured resistance exercise training.

## Conclusion

Though it would be considered a significantly less catabolic stimulus than bed rest, SR as a model of reduced activity results in marked negative alterations in skeletal muscle health in younger and older adults. Periods of SR may occur at increased frequencies in comparison to complete unloading and with, we speculate, underappreciated consequences. In younger persons such periods may not be as deleterious as in older persons since, even given the small number of observations, it appears that older persons have difficulty fully recovering from SR or disuse. We propose that the periodic effects of muscle disuse and SR and the cumulative negative consequences that should be considered in addressing the longer-term health of aging individuals. These periods accelerate muscle loss and induce metabolic dysfunction from which for older persons would have deleterious consequences. Resistance exercise, even low load and sporadically performed, may serve as an effective strategy to offset disuse induced losses in skeletal muscle with SR. Importantly, resistive exercise combined with nutritional stimuli (high quality protein, creatine, and n-3 fatty acids, energy balance) may aid in attenuating the decline in LBM with disuse and aid in the rehabilitation of muscle mass. Given, that nutritional modification or RT is not always feasible during disuse, future research should aim to examine how to improve the recovery period from SR using exercise, rehabilitation, or supplementation to improve the physiological decline with disuse.

## Author Contributions

SO prepared the original draft of the manuscript. SO, TH, and SP contributed to the editing and preparation of the final manuscript.

### Conflict of Interest Statement

SP declares that he has received competitive grant funding, honoraria, and travel expenses from the US National Dairy Council and the Dairy Farmers of Canada. The remaining authors declare that the research was conducted in the absence of any commercial or financial relationships that could be construed as a potential conflict of interest.
